# Glacial cycles drive rapid divergence of cryptic field vole species

**DOI:** 10.1002/ece3.5846

**Published:** 2019-11-23

**Authors:** Nicholas K. Fletcher, Pelayo Acevedo, Jeremy S. Herman, Joana Paupério, Paulo C. Alves, Jeremy B. Searle

**Affiliations:** ^1^ Department of Ecology and Evolutionary Biology Cornell University Ithaca NY USA; ^2^ Instituto de Investigación en Recursos Cinegéticos, IREC (UCLM‐CSIC‐JCCM) Ciudad Real Spain; ^3^ Department of Natural Sciences National Museums Scotland Edinburgh UK; ^4^ CIBIO/InBio, Centro de Investigação em Biodiversidade e Recursos Genéticos Universidade do Porto, Campus de Vairão Vairão Portugal; ^5^ Departamento de Biologia Faculdade de Ciências Universidade do Porto Porto Portugal

**Keywords:** environmental niche modeling, genetic drift, genotyping‐by‐sequencing, glacial refugia, *Microtus agrestis*, speciation

## Abstract

Understanding the factors that contribute to the generation of reproductively isolated forms is a fundamental goal of evolutionary biology. Cryptic species are an especially interesting challenge to study in this context since they lack obvious morphological differentiation that provides clues to adaptive divergence that may drive reproductive isolation. Geographical isolation in refugial areas during glacial cycling is known to be important for generating genetically divergent populations, but its role in the origination of new species is still not fully understood and likely to be situation dependent. We combine analysis of 35,434 single‐nucleotide polymorphisms (SNPs) with environmental niche modeling (ENM) to investigate genomic and ecological divergence in three cryptic species formerly classified as the field vole (*Microtus agrestis*). The SNPs demonstrate high genomic divergence (pairwise *F*_ST_ values of 0.45–0.72) and little evidence of gene flow among the three field vole cryptic species, and we argue that genetic drift may have been a particularly important mechanism for divergence in the group. The ENM reveals three areas as potential glacial refugia for the cryptic species and differing climatic niches, although with spatial overlap between species pairs. This evidence underscores the role that glacial cycling has in promoting genetic differentiation and reproductive isolation by subdivision into disjunct distributions at glacial maxima in areas relatively close to ice sheets. Future investigation of the intrinsic barriers to gene flow between the field vole cryptic species is required to fully assess the mechanisms that contribute to reproductive isolation. In addition, the Portuguese field vole (*M. rozianus*) shows a high inbreeding coefficient and a restricted climatic niche, and warrants investigation into its conservation status.

## Graphical abstract

We combine analysis of 35,434 single‐nucleotide polymorphisms (SNPs) with environmental niche modeling (ENM) to investigate genomic and ecological divergence in three cryptic species formerly classified as the field vole (*Microtus agrestis*). The genomic data demonstrate divergence and little evidence of gene flow among the three field vole cryptic species, and we argue that genetic drift may have been a particularly important mechanism for divergence in the group. The ENM reveals three areas as potential glacial refugia for the cryptic species and differing climatic niches, although with spatial overlap between species pairs, underscoring the role that glacial cycles play in driving divergence between these species.

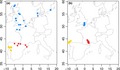

## 1 INTRODUCTION

Genetic variation in contemporary taxa is often heterogeneous across space, with a patchwork of genetically distinct lineages that have been shaped by various historical and ecological processes (Avise, 2000; Moritz, 1994). These processes not only determine the geographic distribution of the entities that make up a species but also mold their adaptive potential and evolutionary trajectories (Funk, McKay, Hohenlohe, & Allendorf, 2012; Moritz, 1994; Waples, 1991). Genetically divergent lineages are the precursors of new reproductively isolated forms and can be considered the true origin of species (Broughton & Harrison, 2003; Feder, Egan, & Nosil, 2012).

In the case of cryptic species, that is two or more similar species that were previously conspecific and essentially indistinguishable in gross morphology, the assessment of genetic differentiation is the foundation of their discovery (Bickford et al., 2007; Sáez & Lozano, 2005). Cryptic species complexes form a critical, yet understudied, component of biodiversity and while (by definition) they are morphologically similar, life history, ecological, or behavioral differences may impact their role in the ecosystem and their susceptibility to extirpation or need for conservation (Bickford et al., 2007; Geller, 1999; Ravaoarimanana, Tiedemann, Montagnon, & Rumpler, 2004). Behavioral differences may also allow members of cryptic species to distinguish their own kind through nonvisual communication such as electrophysiological, auditory, or chemical cues (e.g., Cardé et al., 1978; Feulner, Kirschbaum, Schugardt, Ketmaier, & Tiedemann, 2006; Narins, 1983). The genetic distinctiveness that allows cryptic species to be identified may be substantial, driven by the same processes of isolation and accumulation of genetic differences as in “noncryptic” species complexes (Paupério et al., 2012; Sanders, Malhotra, & Thorpe, 2004), with implications for their ecological and evolutionary potential (Geller, 1999; Ravaoarimanana et al., 2004).

Genetic divergence between contemporary cryptic species may have been driven by geographic isolation during Pleistocene glacial maxima (e.g., Haffer, 1969; Lovette, 2005; Rand, 1948; Stewart, Lister, Barnes, & Dalén, 2010). Europe offers a particular opportunity to study cryptic speciation due to close proximity of ice sheets at glacial maxima, and geographic complexity of the region that resulted in multiple independent glacial refugia and subsequent northward expansions during interglacials (Hewitt, 2000; Stewart et al., 2010). Populations in different refugia would have experienced different selective pressures during isolation that, along with genetic drift, would have promoted genetic divergence (Stewart et al., 2010). The amount of genomic differentiation between separated refugial populations may reflect a variety of evolutionary processes, including but not limited to: local adaptation and divergent selection during isolation, the amount of gene flow between the refugia, and the extent of historic reductions in population size (Funk et al., 2012; Kohn, Murphy, Ostrander, & Wayne, 2006; Thomas & Klaper, 2004). Genetic drift is especially likely to have been important in refugia where population sizes were small enough to overwhelm the forces of natural selection (Stewart et al., 2010). These multiple microevolutionary processes created the opportunity for accelerated speciation during the glacial cycles (Weir & Schluter, 2004).

The integration of genomic analyses and environmental niche modeling (ENM) may allow a powerful investigation of cryptic speciation during the Pleistocene. In the absence of direct observations, genomic analyses can serve as essential proxies for assessing both historic differentiation and current reproductive isolation between forms (Bickford et al., 2007; Kohn et al., 2006; Thomas & Klaper, 2004). Additionally, ENM provides a valuable assessment of the ecogeographical gradients that limit a species' distribution, providing insight into the aggregate environmental and geographical spaces of the species that may be important in distinguishing characteristics between cryptic species. Moreover, ENM can provide the information needed to generate and test hypotheses concerning the structure of genetic diversity that can be relevant to evaluate hypotheses of glacial refuges (e.g., Richards, Carstens, & Knowles, 2007).

The system of cryptic speciation that we analyze here is within the vole genus *Microtus*. This genus includes more than 65 species that originated roughly 2 million years ago (based on a robust fossil record), indicating that *Microtus* has an exceptionally high speciation rate, ~60–100 times higher than typical vertebrates (Chaline, Brunet‐Lecomte, Montuire, Viriot, & Courant, 1999; Triant & DeWoody, 2006). This speciation rate is matched in only a few other vertebrate groups, perhaps most notably the African cichlids (Brawand et al., 2014). Unlike cichlids, whose rapid radiation has been attributed to selection at multiple regions of the genome (Brawand et al., 2014), the mechanisms underlying the high speciation rate in *Microtus* are poorly known. The genus has a Holarctic distribution and many species occur in areas abutting the maximum glacial extent (Jaarola et al., 2004). Therefore, the *Microtus* radiation offers an intriguing system to study complementary and potentially different mechanisms for rapid speciation compared to other classic vertebrate models of speciation.

In particular, the traditional Eurasian “field vole *Microtus agrestis*” (Wilson & Reeder, 2005) includes three major evolutionary lineages that split very recently, are morphologically and karyotypically similar, yet show up to 6% divergence at cytochrome *b* and reciprocal monophyly at multiple nuclear loci (Giménez, Paupério, Alves, & Searle, 2012; Jaarola & Searle, 2004; Paupério et al., 2012). Following Paupério et al. (2012) and Kryštufek (2017), these three divergent lineages will be considered cryptic species: The newly named Portuguese field vole (*M. rozianus*) and Mediterranean field vole (*M. lavernedii*), with the most widespread form, the short‐tailed field vole, retaining *M. agrestis* as a Linnean name. According to Paupério et al. (2012), the Portuguese lineage became separated from the combined Mediterranean and short‐tailed field vole lineages around ~70,000 years ago, at the maximum glacial extent in northern Iberia, while the latter two lineages subsequently originated during the Last Glacial Maximum (LGM) in Europe ~25,000 years ago. The Portuguese and Mediterranean cryptic species have relatively restricted distributions within the Iberian Peninsula and Southern Europe, while the short‐tailed field vole is present in much of Eurasia, from Britain and France in the west to central Siberia in the east. Within the short‐tailed field vole, six genetic lineages split during the Younger Dryas ~ 12,000 years ago and subsequently expanded to fill this range (Herman & Searle, 2011). The dates for these splits are based on robust mtDNA molecular clock rate calibrated from the dates of postglacial ice sheet retreat and sea level rise that limited the potential time of colonization of Britain (Herman et al., 2014) and consistent with ancient DNA analysis of another *Microtus* species (Martínková et al., 2013). Therefore, the field vole cryptic species complex includes a hierarchy of differentiation caused by lineage splits associated with different climatic events and locations during the Late Pleistocene, making it an excellent system to understand the basis of rapid speciation and the role of recent glacial cycles in population divergence and speciation.

**Figure 1:**
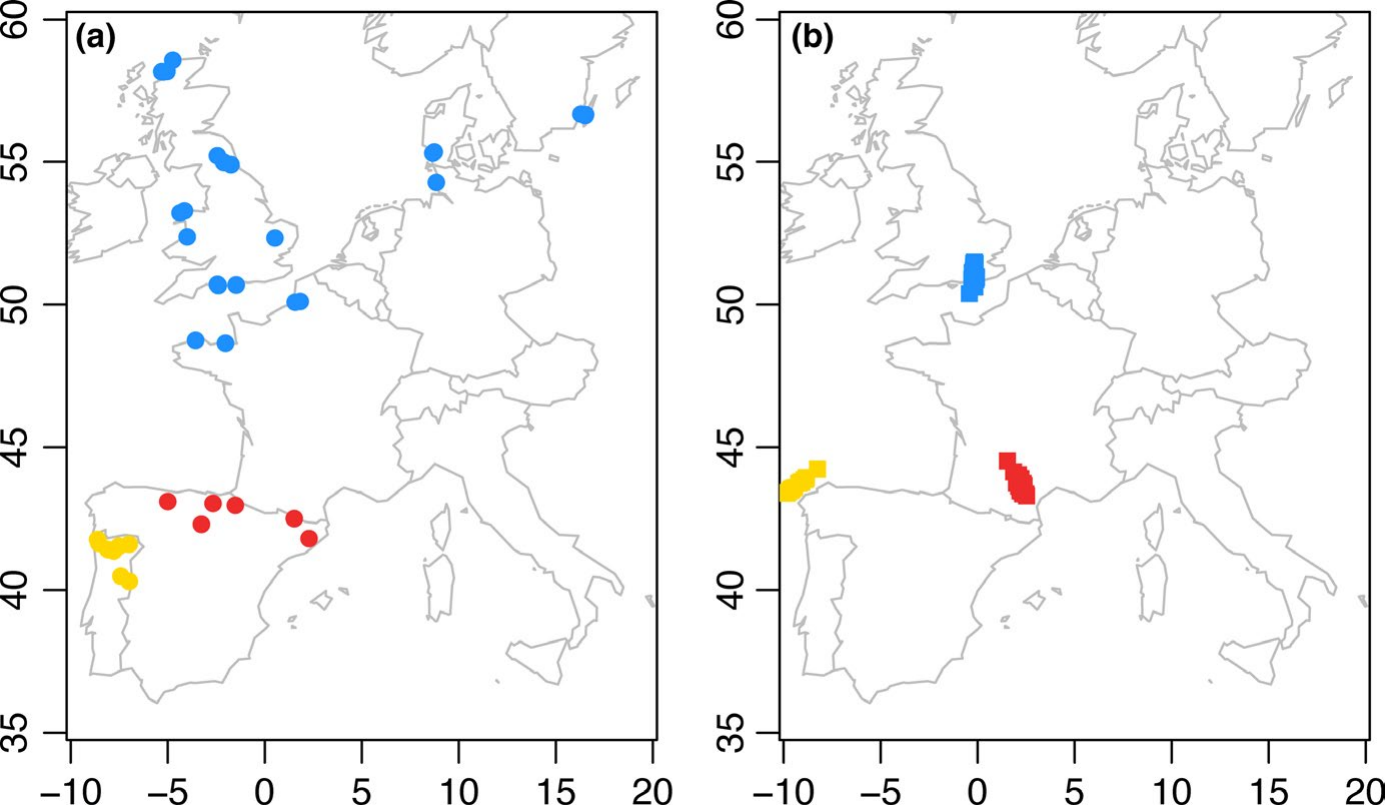
(a) Western European sampling localities for the three species included in SNP analyses. (b) Procrustes transformed genetic and geographic coordinates (details of procrustes transformation in methods). Portuguese (yellow), Mediterranean (red), and short‐tailed (blue) field voles

In this study, we investigated the rapid divergence of the field vole cryptic species by using genotyping‐by‐sequencing (GBS; Elshire et al., 2011) to sequence anonymous single‐nucleotide polymorphisms (SNPs) across the genome of 83 individuals. Our sampling includes individuals of all three field vole cryptic species from western Europe (Figure 1a, Table S1). We aim to characterize the level of genomic divergence that has led to the formation of cryptic species within the last glacial cycle, presumably driven by geographic isolation in glacial refugia. In order to assess the putative climatic basis of differentiation, we performed ENM to estimate the climate favorability for the field vole at Last Glacial Maximum (LGM) and, finally, we studied the biogeographic relationships among its cryptic species at present. Using these analyses, we characterized broad‐scale genomic and ecological differentiation among the three field vole cryptic species and confirm the high levels of both genomic divergence and differences in their climatic envelope. These analyses contribute to our understanding of how isolation during glacial cycling can cause rapid, yet high genome‐wide divergence between populations that result in new (cryptic) species.

## 2 METHODS

### 2.1 DNA extraction and genotyping‐by‐sequencing

Genomic DNA was extracted from ethanol‐preserved vole tissues (ears or digits) from 83 specimens, collected between 1995 and 2009 in western Europe (Figure 1a, Table S1), using the DNeasy Blood and Tissue kit (Qiagen) following standard protocols. GBS was conducted on extracted DNA by the Cornell Institute for Genomic Diversity (Elshire et al., 2011). The enzyme PstI (CTGCAG) was used for digestion, and the fragmented DNA was then ligated to a bar‐coded adaptor and a common adaptor with the appropriate “sticky ends.” Each individual was given a unique barcode combination and, after ligation, all individuals were pooled into a single Eppendorf tube. The libraries were then subjected to PCR, using primers that matched the barcoded and common adaptors, to amplify appropriately sized sequence fragments and to add sequencing primers to the libraries. Libraries were cleaned using a Qiaquick PCR Purification Kit (Qiagen). They were sequenced using single‐end 100 bp reads on two separate lanes of an Illumina HiSeq 2000 at Cornell University Life Sciences Core Laboratories Center.

### 2.2 SNP genotyping and filtering

Raw FASTQ files from the Illumina run were converted to individual SNP genotypes using the TASSEL GBS pipeline, as part of the TASSEL 5.0 software (Bradbury et al., 2007). We implemented the standard TASSEL pipeline with the following parameters. First, we found all reads with barcodes that match the index file and trimmed them to 64 bp to create tags. Only tags with a minimum number of five reads were retained and subsequently merged into a master tag list for each individual. Only tags with one SNP per fragment were included in this analysis. All reads were aligned to the *Microtus ochrogaster* reference genome (McGraw, Davis, Young, & Thomas, 2011) using BWA (Li & Durbin, 2009). The error rate for SNP calling was set to 0.03, with a genotypic mismatch rate set to 0.1. To minimize sequencing error, triallelic minor alleles were excluded, as well as SNPs with a minor allele frequency < 0.05. Only SNPs that were present in >60 of 83 individuals (~72%), and therefore shared across all three cryptic species, were included. It should be noted that changing this missing data threshold to 80%, 90%, and 100% did not change the pattern of our results (data not shown). Individuals with a proportion of missing SNP data >0.25% were excluded, and the average proportion of missing SNP data per individual was 0.066% across all individuals after filtering. Data were converted to vcf files for subsequent analyses. To filter out SNPs from paralogous loci, SNPs were excluded if they showed significant (*p* < .05) excess heterozygosity and deviation from Hardy–Weinberg equilibrium as calculated using vcftools (Danecek et al., 2011).

### 2.3 Genetic diversity patterns

Weir and Cockerham's *F*_ST_ and the inbreeding coefficient F were calculated using *vcftools* (Danecek et al., 2011). To visualize genetic clusters, principal component analysis (PCA) was performed with the TASSEL 5.0 software using standard parameters (Elshire et al., 2011). File conversion for subsequent analyses was done using the software PGDspider (Lischer & Excoffier, 2012). To examine patterns of genetic differentiation and admixture, we used STRUCTURE version 2.3 with a 50,000‐cycle run burn‐in period followed by 50,000 cycles using an admixture model and correlated allele frequencies among groups (Pritchard, Stephens, & Donnelly, 2000). This was repeated for *K* = 1–5. STRUCTURE HARVESTER (Earl, 2012) was used to determine the *K* with highest log‐likelihood. Data outputs were visualized using custom R scripts (R Development Core Team, 2008) included on our Data Dryad submission. To investigate if genome‐wide differentiation patterns are driven by geographic sampling of the individuals, we compared genomic PCA distributions to geographic distributions using a Procrustes transformation technique (Wang et al., 2010). The first two PCA eigenvectors and the latitude and longitude of the individual sample locations were superimposed, with the PC axes rotated to maximize similarity to the geographic distribution of sampled locations. The significance of the association was evaluated based on 10,000 permutations. Procrustes transformations and maps were implemented in R using the “MCMCpack” and “vegan” packages (R Development Core Team, 2008).

### 2.4 Environmental niche modeling

Environmental niche modeling can provide information needed to understand the past structure of genetic diversity (e.g., Richards et al., 2007), supplying complementary information to that obtained from phylogeography (e.g., Diniz‐Filho, Gouveia, & Lima‐Ribeiro, 2013). In this context, we used ENM to: (a) Parameterize a model to be hindcasted for the LGM to make inferences about glacial refugia for the species and (b) parameterize models for each cryptic species of field vole to determine their biogeographical relationships.

#### 2.4.1 Glacial refugia

The starting point for these analyses was distribution data from the Atlas of European Mammals (Mitchell‐Jones et al., 1999) where the field vole is reported in 1,439 UTM 50 km × 50 km (out of the 2,553 squares in western Europe). Analyses were restricted to western Europe in order to exclude the most incomplete sampling areas identified by the authors of the Atlas (A. J. Mitchell‐Jones, personal communication). Fløjgaard, Normand, Skov, and Svenning (2009) studied the main environmental gradients constraining the species distribution range and showed that a model including water balance and growing degree‐days provided the best predictive ability for the occurrence of the field vole (AUC = 0.802) that was closely followed by a model based on annual mean temperature and annual precipitation (AUC = 0.766). However, in revisiting their data for western Europe, the two models are virtually equivalent (only when considering Siberia did the models differ). Therefore, given the interpretability of the predictor variables and the restriction to western Europe of our approach, we feel justified in the present study to parameterize a model including annual mean temperature (BIO1) and annual precipitation (BIO12) as climatic predictors, obtained from the Worldclim project (http://www.worldclim.org/bioclim; Hijmans, Cameron, Parra, Jones, & Jarvis, 2005). Accordingly, four models using the selected predictors were compared (quadratic terms were estimated by centring the variables on the mean, and they were included in order to account for nonlinear responses of the species to these climatic gradients):

model.1: ~BIO1 + BIO12.
model.2: ~BIO1 + BIO1^2^ + BIO12.
model.3: ~BIO1 + BIO12 + BIO12^2^.
model.4: ~BIO1 + BIO1^2^ + BIO12 + BIO12^2^.

For ENM, the geographic area of analysis was first delimited using a Trend Surface Analysis (TSA; see details in Acevedo, Jiménez‐Valverde, Lobo, & Real, 2012). Then, the dataset with the localities selected after TSA was split and each model was parameterized on the training dataset (80%) and its predictive performance was evaluated on the validation dataset (20%). For each model, logistic regression was used to relate the predictors with species distribution range since it has proved to produce consistent results when models are hindcasted to past climatic scenarios (Acevedo et al., 2015). Their performance was assessed on the validation dataset both in terms of discrimination (AUC) and reliability (Hosmer and Lemeshow test, and calibration plots), the latter being more informative of their predictive capacity (see Jiménez‐Valverde, Acevedo, Barbosa, Lobo, & Real, 2013). Models were compared in terms of predictive performance, and the best model was selected. The selected model was then transferred by projecting the model on bioclimatic variables for the LGM (Braconnot et al., 2007). For this purpose, we considered the CNRM‐CM5 Global Circulation Model for models transference. Previously, as the models are not able to accurately predict beyond the range of values of the predictors used for training (Werkowska, Márquez, Real, & Acevedo, 2016), a multivariate environmental similarity surface (MESS) analysis was developed (Elith, Kearney, & Phillips, 2010). Therefore, the model was not projected to the past in those squares outside the climatic range represented in the training dataset.

#### 2.4.2 Biogeographical relationships

To determine the biogeographical relationships between cryptic species, we constructed a dataset including the distributions of the cryptic species based on 377 voles previously assessed using diagnostic mtDNA and nuclear markers sampled throughout the range (Portuguese [P] = 67 localities, Mediterranean [M] = 84 and short‐tailed [St] = 226, data from: Hellborg, Gündüz, & Jaarola, 2005; Herman & Searle, 2011; Herman et al., 2014; Jaarola & Searle, 2002; Jaarola & Searle, 2004; Paupério et al., 2012, J. Paupério, unpublished data). The first stage was to model, at locality level, pairs of adjacent cryptic species (i.e., P vs. M and M vs. St), using bioclimatic variables as predictors, in order to obtain models able to discriminate between each pair of species. The probabilities of species presence were then projected to the entire species distribution range and used to assign a particular cryptic species to each 50 km × 50 km UTM squares with presence of the field vole (Mitchell‐Jones et al., 1999); for this purpose, the threshold that minimizes the difference between sensitivity and specificity was used (Liu, White, & Newell, 2013).

In the next stage, the distribution of each cryptic species in western Europe at 50 × 50 km UTM square resolution was modeled. This procedure permitted a more complete range of species distributions in the ENMs, avoiding the sampling effort bias that would be included when directly modeling raw data at a sampling locality scale. To obtain the 50 × 50 km UTM square resolution, we used the presence/absence data predicted in the previous stage. Modeling each cryptic species independently can result in an incomplete environmental response of the species to climatic conditions (e.g., Sánchez‐Fernández, Lobo, & Hernández‐Manrique, 2011). Thus, a new model for each cryptic species was parameterized including just the predictors selected in the previous model including the overall species distribution plus the spatial factor (longitude and latitude) to account for the current spatial range in the predictions. Models for each cryptic species were developed using logistic regressions.

Finally, to establish direct comparisons between ranges of cryptic species distribution, the logistic probabilities were included in the favorability function described by Real, Barbosa, and Vargas (2006). The favorability function is a valuable tool to study biogeographical relationships between models whatever the sample prevalence in the calibration datasets (e.g., see Acevedo and Real, 2012). The biogeographical relationships between two cryptic species were assessed from the favorabilities and using the fuzzy overlap index (FOvI; see details Acevedo, Ward, Real, & Smith, 2010), that is, the ratio between the degree to which the study area is favorable to the two studied cryptic species simultaneously and the degree to which it is favorable for either species (Dubois & Prade, 1980; Kunchenva, 2001). This index varies from 0 (no overlap in favourability) to 1 (complete overlap in favorability). The FOvI can be decomposed into absolute local overlap values (FOvI‐L) that represent the contribution of each locality (Universal Transverse Mercator (UTM) square) to the FOvI. Thus, the FOvI‐L shows the spatial location of the areas where spatial overlap between species is expected to occur (Acevedo et al., 2010). According to these authors, three thresholds in FOvI‐L can be defined: FOvI‐L < 0.2 autoecological segregation, 0.2 > FOvI‐L > 0.8 competitive exclusion, and FOvI‐L > 0.8 coexistence. Absolute local overlap values indicate the minimum environmental favorability that a given locality achieves for the two species, and whether one is predicted to have higher favorability than the other. These analyses of biogeographical relationship between interacting species were carried out with the favorability values derived for ENM reported in Figure S5.

## 3 RESULTS

### 3.1 Sequence data quality control and filtering

We sequenced a total of 198,371,000 reads across 83 individuals. Of these, 166,336,045 reads contained unique barcodes and cut sites, and contained no “N”s. These reads corresponded to 692,765 tags across all individuals that met the minimum 5× threshold coverage. Of these tags, a total of 338,732 could be aligned to unique positions in the *M. ochrogaster* genome. After filtering for missing data, minor allele frequency and excess heterozygosity, there were 35,434 loci, with an average of 6.55% missing data across all sites and individuals.

### 3.2 Genetic diversity patterns

Global *F*_ST_ among the three cryptic field vole species, using the 35,434 loci, was 0.453 and the frequency distribution of *F*_ST_ across sites is shown in Figure S1. Weir and Cockerham's weighted pairwise *F*_ST_ estimates between cryptic species pairs and the average inbreeding coefficient (*F*) are summarized in Table 1. Pairwise *F*_ST_ was highest between the Portuguese and other cryptic species of field vole (vs. Mediterranean = 0.72; vs. short‐tailed = 0.69). There was a somewhat lower pairwise *F*_ST_ = 0.45 between the Mediterranean and short‐tailed cryptic species. The average inbreeding coefficient was distinctly higher for the Portuguese cryptic species (*F* = 0.75) than the others (Table 1).

**Table 1:** Average individual inbreeding coefficient (*F*) and Weir and Cockerham's weighted pairwise *F*_ST_ for the Portuguese, Mediterranean, and short‐tailed cryptic species of the field vole

	*F*	*SD*	Portuguese *F*_ST_	Mediterranean *F*_ST_	Short‐tailed *F*_ST_
Portuguese	0.75	0.033	0.00		
Mediterranean	0.63	0.068	0.72	0.00	
Short‐tailed	0.60	0.090	0.69	0.45	0.00

**Figure 2:**
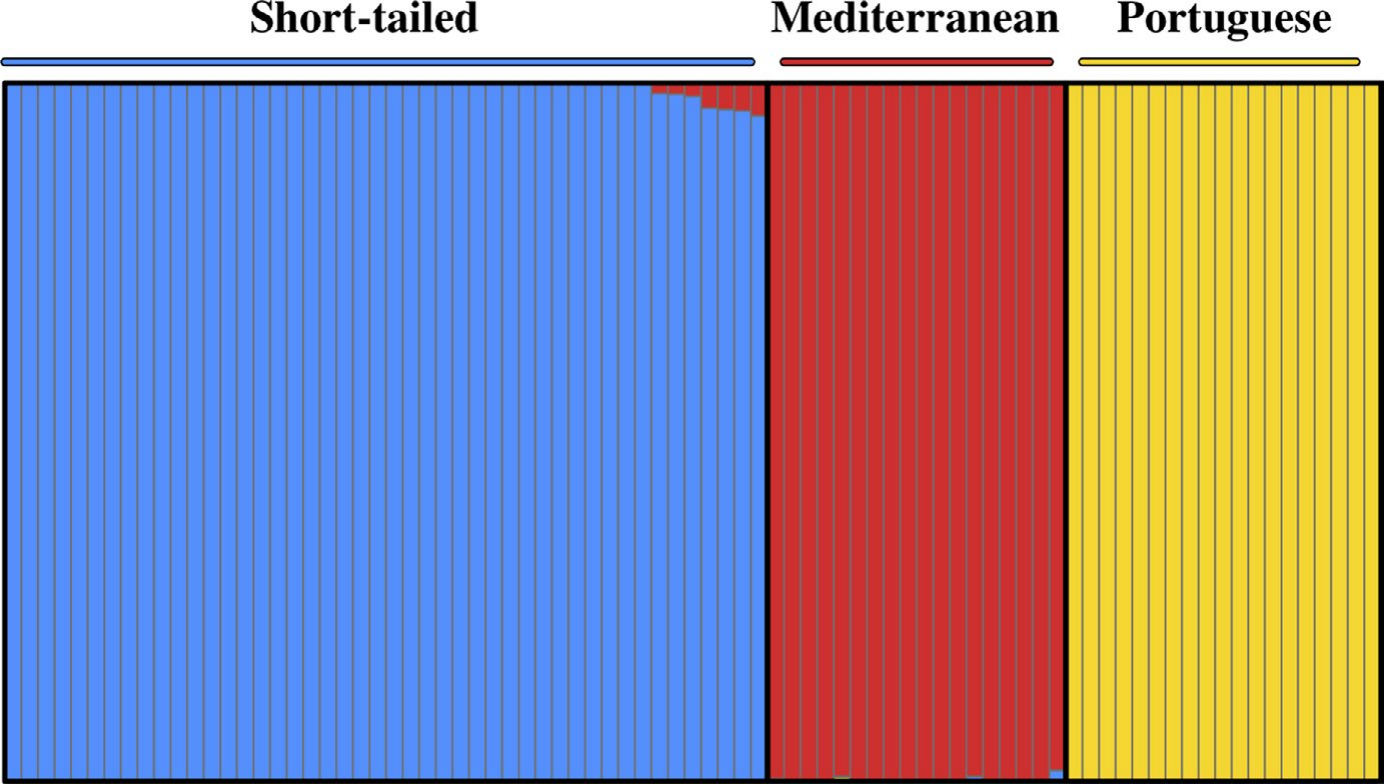
STRUCTURE plot showing *K* = 3 for short‐tailed (blue), Mediterranean (red), and Portuguese (yellow) field voles

**Figure 3:**
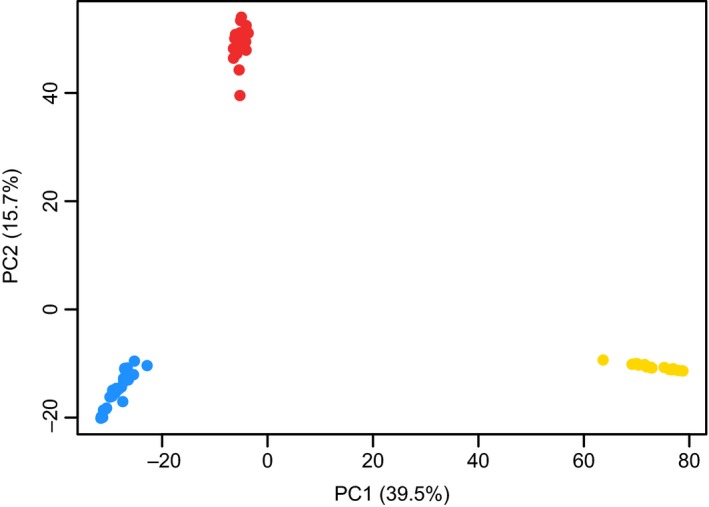
PCA summarizing 35,434 SNP loci for Portuguese (yellow), Mediterranean (red), and short‐tailed (blue) field vole individuals

Our STRUCTURE analysis of 35,434 loci supported *K* = 3, as Structure Harvester showed highest Δ*K* log‐likelihood change from *K* = 2 to *K* = 3 as per the Evanno, Regnaut, and Goudet (2005) method. The most probable *K* reflected the distribution of our three predicted cryptic species. The STRUCTURE clusters are composed of populations with extremely low admixture between the short‐tailed and Mediterranean cryptic species of field vole, and no admixed individuals with both Mediterranean and Portuguese cryptic species ancestry (Figure 2). These results are supported by PCA analysis; clear separation of all three cryptic species, with tight clustering of individuals within each species (Figure 3). PC1 explained 39.5% of the variation among individuals and the PC1 axis clearly separated the Portuguese cryptic species from the Mediterranean and short‐tailed. PC2 explained 15.7% of the variation among individuals, and the Mediterranean cryptic species was distinctive along this axis. The rotated genetic coordinates shown graphically in the Procrustes analyses revealed that the individual cryptic species cluster more tightly by refugial origin than would be predicted by geography alone (Figure 1b).

### 3.3 Environmental niche modeling

#### 3.3.1 Glacial refugia

**Figure 4:**
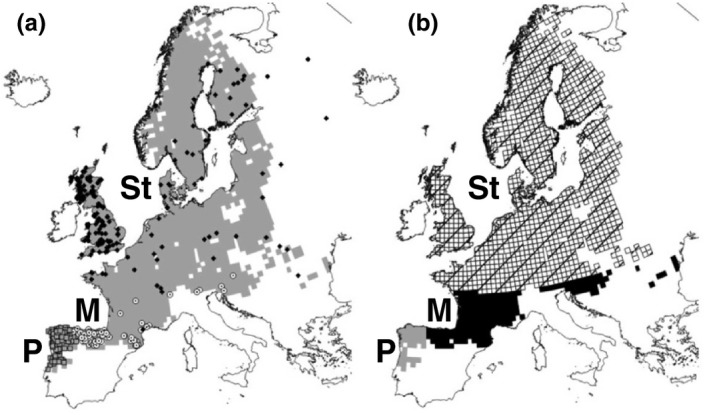
(a) Individuals used for parameterizing climatic niche models (*N* = 377) and the distribution of cryptic species of field vole [P: Portuguese (gray squares), M: Mediterranean (white circles), and St: short‐tailed (black diamonds)] in western Europe (our area for modeling; see text for details). Area in gray shows the field vole distribution in western Europe (Mitchell‐Jones et al., 1999). (b) Predicted cryptic species distribution in Europe according to environmental niche modeling (see text for details) and the species distribution recorded by Mitchell‐Jones et al. (1999). Hatching = short‐tailed, solid black = Mediterranean, and solid gray = Portuguese. Grid cells represent the UTM 50 × 50 km squares

**Figure 5:**
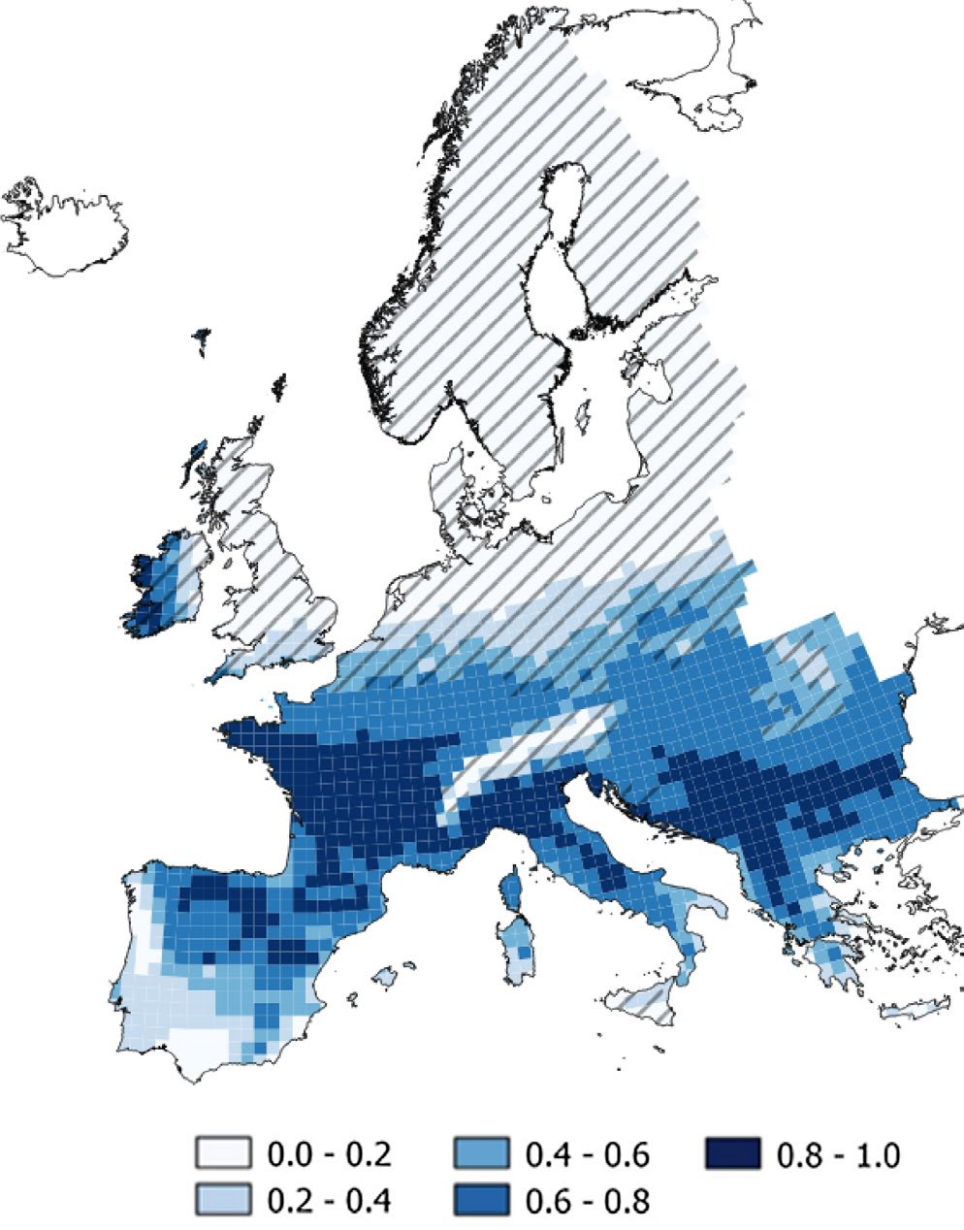
Predicted probability of occurrence of the field vole during the Last Glacial Maximum (LGM) in western Europe according to climatic niche model 4 (individual cryptic species not identified). Hatched areas were excluded for model transferability according to a MESS analysis. Grid cells represent the UTM 50 × 50 km squares

The bioclimatic predictors showed significant effects in all niche models (Table S2). AICs for the models, their predictive performances and a cartographic representation of the predicted probabilities are shown in Figure S3. Model.4 was selected based on explanatory capacity and predictive performance. After MESS, model.4 was projected onto the LGM only in the areas within its environmental domain (Figure 4). Predictions for the LGM can be used to delimit potential refugia for the species (predicted *p* > .8; see Figure 5).

#### 3.3.2 Biogeographical relationships

**Figure 6:**
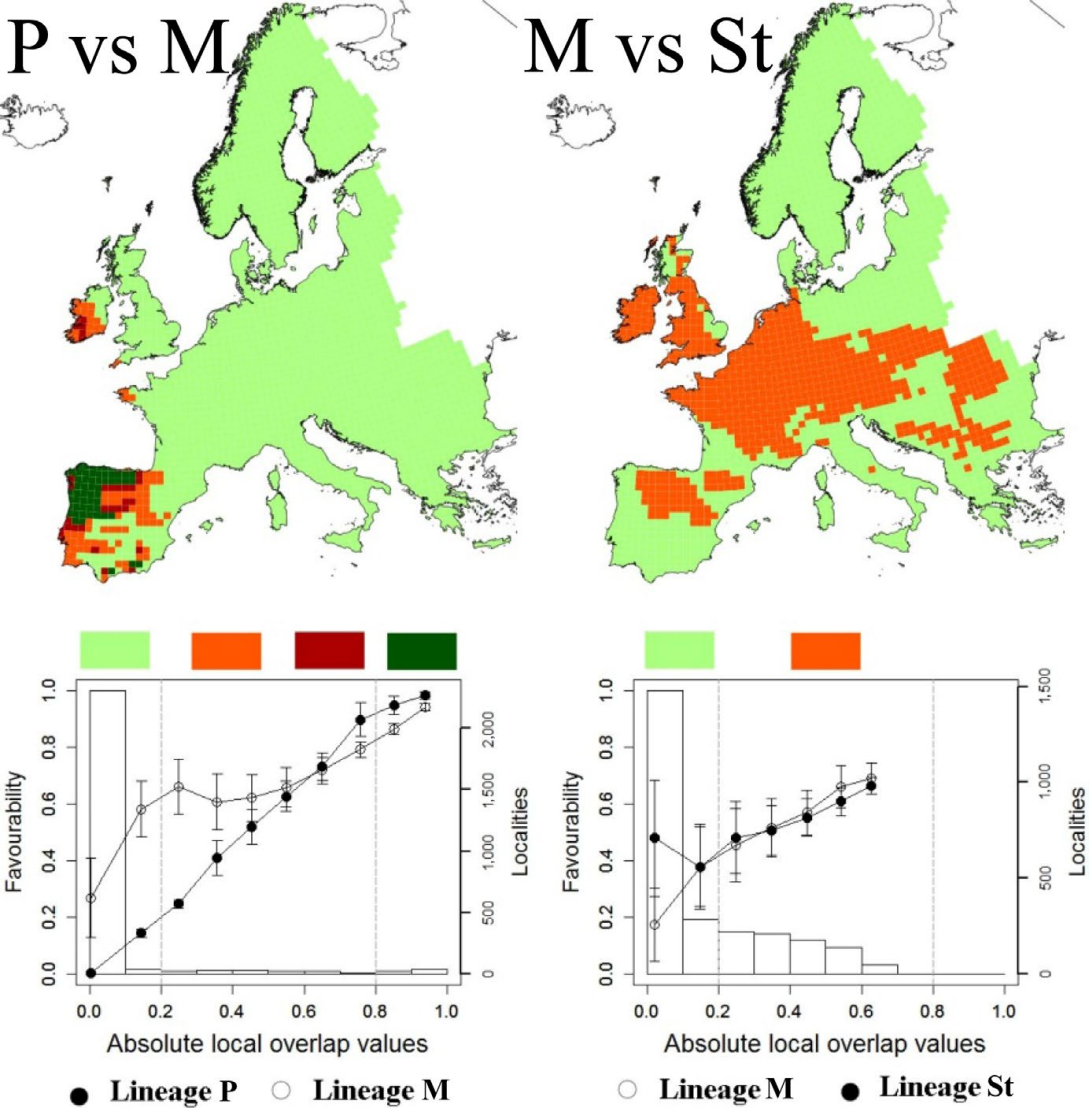
Biogeographical relationships between the cryptic species of the field vole. (P: Portuguese, M: Mediterranean, and St: short‐tailed). Mean favorability scores (defined for each species) are plotted against gradients defined by the absolute local overlap values. The gradients were divided in natural intervals, and mean favorability values (±*SD*) are shown. The number of sampling sites at each interval is also shown in columns. Areas with higher local overlap of favorability scores between species increase on the *x*‐axis. Fixed intervals are defined (dotted vertical lines) in the charts and mapped, with light green being the lowest, orange and maroon being median, and dark green being the highest local overlap. Grid cells represent the UTM 50 × 50 km squares

The models at locality level (see Table S3) discriminated well between the Portuguese and Mediterranean cryptic species of field vole (AUC: 0.999 and H‐L: 5.78, *p* > .05) and between Mediterranean and short‐tailed cryptic species (AUC: 0.999 and H‐L: 10.94, *p* > .05). These models were used to assign a cryptic species to each UTM square (Figure 5). Results of the subsequent independent models for each species distribution at UTM square level are shown in Table 2 (see also Figure 6). Models showed that the climatic factors modulating the species distribution at a global scale are affecting each cryptic species differently (Table 2).

**Table 2:** Statistical parameters for validation (discrimination, area under the curve [AUC]; reliability, Hosmer and Lemeshow test [H–L]), as well as individual predictors (AUC/H–L) of the climatic niche models for the Portuguese, Mediterranean, and short‐tailed cryptic species of the field vole

	Portuguese	Mediterranean	Short‐tailed
Parameter			
Model	~BIO1‐BIO1^2^ + BIO12‐BIO12^2^ ‐X‐Y	~‐BIO1‐BIO1^2^ + BIO12‐BIO12^2^ ‐X‐Y	~‐BIO1‐BIO1^2^‐BIO12‐BIO12^2^ ‐X + Y
AUC	0.995	0.888	0.882
H‐L	4.21 n.s	12.46 n.s.	16.14*
Predictor			
BIO1	1.2e−01/2.04*	−3.9e−03/−0.61 n.s.	−3.6e−02/−9.32***
BIO1^2^	−2.2e−03/−3.55***	−5.3e−04/−6.02***	−5.9e−04/−15.56***
BIO12	5.2e−03/3.07**	3.8e−03/7.54***	−2.7e−03/−7.58***
BIO12^2^	−1.1e−05/−2.94**	−16e−06/−5.12***	−7.8e−07/−1.76n.s.
Longitude	−1.0/−4.62***	−7.2e−02/−7.70***	−6.4e−02/−8.38***
Latitude	−6.5e−01/−5.22***	−3.2e−01/−12.06***	1.2e−01/9.20***
(Intercept)	1.7e + 01/1.90*	1.2e + 01/7.90***	9.3e−01/0.82 n.s.

Abbreviation: n.s., not significant.

* *p* < .05.

** *p* < .01.

*** *p* < .001.

The biogeographical relationships between cryptic species are shown in Figure 6. The results suggest the existence of zones of potential co‐occurrence in most of the core area of the distribution of the Portuguese cryptic species. Areas where the Portuguese cryptic species could be excluded by the Mediterranean are limited and currently distributed close to the edge of the Portuguese cryptic species distribution. By contrast, areas of coexistence for the Mediterranean and short‐tailed cryptic species are not predicted by the models. Predicted areas of coexistence are those in which the environmental conditions are highly favorable for both species. The scarcity of the predicted areas of coexistence for the short‐tailed and Mediterranean cryptic species is explained by the small area in which the two species overlap and interact, being in localities that are far from each of their optimal niches and at the edge of their ranges. In addition, even when models show a wide area of potential overlap between the two cryptic species, neither species has high favorability in those overlap areas.

## 4 DISCUSSION

We showed substantial genome‐wide differentiation between the Mediterranean, short‐tailed and Portuguese cryptic species of the field vole, which were until recently considered a single species (Mathias, Hart, Ramalhinho, & Jaarola, 2017). This high genetic differentiation is reflected in their high levels of *F*_ST_ across the genome as well as by the clear separation of clusters in the PCA. The Procrustes analysis comparing separation in PCA space to the geographic separation of individuals sampled confirms that current geographic sampling location alone cannot explain these patterns, and that they cluster instead according to different LGM refugial origin of each cryptic species. Previous studies have shown that although the field vole range extends far east into Siberia, those eastern individuals represent an expansion from a single post‐LGM population and are closely related to voles in northern Scandinavia and central Europe, which are represented in this study (Herman & Searle, 2011). Our sampling was restricted to western Europe to try and pinpoint the environmental factors limiting gene flow between the cryptic species in the area where they all co‐occur. Analyses of short‐tailed field vole populations alone revealed high intraspecific genetic structure, suggesting that even more recent climatic oscillations may play a role in driving divergence within species, including the Younger Dryas cold period (Figure S7, Herman & Searle, 2011). The divergence is genome‐wide across most loci as shown in Figure S1 and is unlikely that it is driven by selection alone. The levels of pairwise *F*_ST_ between the cryptic species of the field vole are extremely high, especially when considering that these lineages have only been separated for 30–70,000 years (Paupério et al., 2012). High levels of genetic drift along with low levels of admixture between the cryptic species are most likely to have driven these divergence patterns. A short generation time (less than 1 year; Mathias et al., 2017) is likely important for the rapid evolution and, importantly, this species is also known for its multi‐year population cycling, which if occurring in isolated populations during refugial isolation, may increase fixation of alternative alleles and drive divergence between lineages (Ehrich, Yoccoz, & Ims, 2009).

Our data also indicate a divergence in the environmental niche among the three cryptic species, although at a lower level than that observed from the genomic data. Interestingly, this is particularly true when comparing the environmental niche between the Portuguese and Mediterranean cryptic species, which shows low divergence despite the genomic differentiation being the highest observed. The short‐tailed cryptic species occupies a relatively cooler and more humid niche than the others, and the Portuguese cryptic species a relatively hotter and more humid niche than the Mediterranean. Although there are differences between the climatic niches of each cryptic species, our models predict a wide geographic area of potential overlap between the Portuguese and Mediterranean cryptic species, but this is not reflected in the contemporary distribution of the genetic lineages. This suggests some pre‐ or postzygotic barrier to reproduction between these groups. These intrinsic barriers to reproduction would promote species boundaries, despite similar external morphologies and climatic niches (Rowe, Aplin, Baverstock, & Moritz, 2011). A recent fine‐scale study on the contact zone between the Portuguese and Mediterranean cryptic species also supports our conclusions, where no hybrid individuals were found and the contact zone is estimated to be less than 15 km wide, despite no obvious geographic or ecological barriers (J. Paupério, unpublished data). Both of these lines of evidence suggest that the Mediterranean and Portuguese cryptic species are essentially reproductively isolated despite less than ~70,000 years of divergence. This is contrasted with the pattern of differentiation we find between the more recently diverged Mediterranean and short‐tailed field voles, which have lower genetic differentiation, but also much less overlap in our models (Figure 6). Although the results suggest that reproductive isolation is acting as a barrier to gene flow and limiting the distribution of each of the species, they may also differ in other aspects of their ecology or behavior that could have led to other axes of niche specialization that are not measured in our environmental niche models.

It has previously been shown that allopatry during glacial refugial periods allows for the accumulation of genetic differences between lineages, including differences that impact reproductive isolation, determining the amount of subsequent gene flow between forms (Barton & Hewitt, 1985; Weir & Schluter, 2004). Reproductive isolation is even more likely to occur when small population sizes in isolated refugia allow for the fixation of genetic variants (including chromosomal rearrangements, but also genic incompatibilities) that affect the fitness of hybrids upon secondary contact (Dion‐Côté & Barbash, 2017; Hewitt, 1996; Rowe et al., 2011; Searle, 1993). Although isolation during glacial cycling and subsequent secondary contact has been suspected as a scenario that promotes rapid speciation (Hewitt, 1996), the particular geographic context matters to determine whether speciation will occur (Klicka & Zink, 1997).

This study elucidates one potential factor that has contributed to speciation in microtine rodents. Voles in the genus *Microtus* represent one of the most speciose genera of rodents (and mammals) in the world, with over 65 species that arose in the last ~2 million years (Chaline et al., 1999; Musser & Carleton, 2005). This suggests a speciation rate that is matched by few other vertebrate groups that are typically attributed to adaptive radiations resulting in rapid divergence in morphology and ecology (Brawand et al., 2014). Many species groups within *Microtus* have distributions relatively close to where ice sheets occur during glacial maxima and isolation during cyclical glacial maxima may have caused divergence, reproduction isolation and ultimately speciation, between these closely related lineages (Chaline et al., 1999; Weir, Haddrath, Robertson, Colbourne, & Baker, 2016). Environmental niche modeling hindcasted to the LGM shows multiple potential refugial areas for the field vole, each population evolving in allopatry: one in the northeastern Iberia (delimited by Ebro river; the Portuguese cryptic species), another in France or northern Italy (the Mediterranean cryptic species), and finally another in the Balkans (short‐tailed cryptic species; Figure 5). These distributions of refugia are in line with previous hypotheses of refugial locations for the field vole (Paupério et al., 2012). The current distribution of these cryptic species of the field vole reflects common phylogeographic breaks in western Europe, which underscores the role that glacial cycles have played in structuring genetic diversity in the region (Taberlet, Fumagalli, Wust‐Saucy, & Cosson, 1998). In particular, the highly divergent Portuguese field vole species represents another mammal endemic to the Iberian Peninsula, a region harboring the highest level of endemicity in Europe, and already home to another endemic vole in the genus (*M. cabrerae*, Baquero & Tellería, 2001; Bilton et al., 1998). The large amount of divergence across the genome between cryptic species of the field vole points to substantial genetic drift and fixation of alternative alleles during isolation in refugial areas.

If the divergence between forms results in changes that promote reproductive isolation, such as fixation of alternative chromosomal arrangements as is seen in many voles in the genus *Microtus* (Mazurok et al., 2001), lineages will continue to remain evolutionarily distinct even after secondary contact. The three cryptic vole species analyzed in this study have the same number of chromosomes, and similar giant sex chromosomes (Giménez et al., 2012), but it is possible that there are undetected chromosomal rearrangements that could have contributed to reproductive isolation. These cryptic vole species may also have fixed genetic differences that result in genetic incompatibilities in hybrid individuals. For example, heterochromatin differences are thought to contribute to reproductive isolation between diverging lineages by triggering genome instability in hybrids, and the field vole is known for its large blocks of heterochromatin repeats, especially on their giant sex chromosomes (Dion‐Côté & Barbash, 2017; Marchal et al., 2004). There could also be other isolating mechanisms in this group, such as pheromones or urinary proteins that are known to be important for species recognition in rodents, or in genes involved with seminal proteins that may result in incompatibilities expressed in hybrids (Logan, Marton, & Stowers, 2008; Payseur & Nachman, 2005).

Subdivision into isolated glacial refugia may be particularly important in the generation of cryptic species complexes (Hope, Speer, Demboski, Talbot, & Cook, 2012; Weir et al., 2016). There may be a lag between genetic and morphological differentiation in systems where there is no strong selection for gross phenotypic divergence, although divergence may occur in other traits important for maintaining reproductive isolation between these cryptic species (Cardé et al., 1978; Fišer, Robinson, & Malard, 2018). This contributes to the growing number of examples of seemingly “nonadaptive” radiations of cryptic species on continents, in which speciation lacks the accompanying morphological differentiation typically epitomized by adaptive radiations (Mathias et al., 2017; Zelditch, Li, Tran, & Swiderski, 2015). However, environmental niche modeling has shown that these voles have indeed diverged in some aspects of their ecology and therefore labeling this radiation as “nonadaptive” does not capture differences in their ecology and physiology that underlie their differences in climatic niche. Other rodent groups, such as the speciose genus *Rattus*, have likewise shown high rates of speciation without obvious morphological differentiation, and this may represent a common trend in rodent groups with rapid speciation (Maestri et al., 2017; Rowe et al., 2011). This trend is already becoming apparent within *Microtus*. A recent study has revealed two highly genetically distinct populations of *M. californicus*, which are also currently considered one population and lack obvious morphological differentiation (Lin et al., 2018). These populations diverged less than 50,000 years ago have no obvious ecological or geographic barriers separating them, and yet show no evidence of nuclear gene flow (Lin et al., 2018).

Cryptic species are often overlooked, yet they are an important component of biodiversity, and the identification of cryptic species can reveal their unique ecological roles and evolutionary potential (Bickford et al., 2007; Geller, 1999). Genomic tools have much potential in the discovery of cryptic species and genomic differences reveal the underlying processes that restrict gene flow and cause differentiation between forms (Funk et al., 2012). The three cryptic species of field vole complex not only differ greatly at a genome‐wide level, but they have diverged in ecological traits that underpin differences in individual climatic niches. Clearly, it would be interesting in the future to link genotype and phenotype and establish the genomic basis of climatic adaptation in these cryptic species.

The field vole is a key primary herbivore and component of the food web in the grasslands where they occur and has been described as “the most important dietary part of more species of predators than perhaps any other mammal due mainly to its numerical abundance” (Mathias et al., 2017). Our study provides further evidence in support of three recently recognized, taxonomically distinct, cryptic species in the field vole (Kryštufek, 2017; Paupério et al., 2012). On these grounds, it is important to understand the basis of ecological and physiological divergence in these cryptic species and the specific role that they have in their respective environments. Environmental modeling shows that the niche of the Portuguese cryptic species is shrinking under current climate change (Figure S6), and this lineage already shows the highest level of inbreeding of any of the cryptic taxa. As recommended in Paupério et al. (2012), further evaluation of the Portuguese cryptic species in particular is needed to examine the future viability of populations in its range, and the putative need to implement conservation measures to preserve this species. This species may be locally adapted to a habitat that is disappearing given current climate change scenarios.

## CONFLICT OF INTEREST

None declared.

## AUTHOR CONTRIBUTIONS

N.K.F. designed and performed research, analyzed data, and wrote the paper. P.A. performed research and analyzed data. J.S.H. helped design research and contributed samples. J.P and P.C.A. contributed samples. J.B.S. helped design research and write the paper. All authors have read and commented on the paper.

### OPEN RESEARCH BADGES


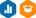
This article has been awarded Open Materials and Open Data Badges. All materials and data are publicly accessible via the Open Science Framework at SNP VCF Dataset: Dryad https://doi.org/10.5061/dryad.zcrjdfn6n; R script for STRUCTURE visualization: https://doi.org/10.5061/dryad.x3ffbg7dw.

## Data Availability

SNP VCF Dataset: Dryad http://doi.org/10.5061/dryad.zcrjdfn6n. R script for STRUCTURE visualization: https://doi.org/10.5061/dryad.x3ffbg7dw. Sampling IDs, locations, and specimen information: Table S1. The data that support the findings of this study will be openly available in Dryad at https://doi.org/10.5061/dryad.zcrjdfn6n.
