# Supplementary figures and images for: Glacial cycles drive rapid divergence of cryptic field vole species
Source: Ecol Evol. 2019 Nov 23;9(24):14101–13. doi: 10.1002/ece3.5846 (PMC6953675; doi:10.1002/ece3.5846)

# Fst across all sites

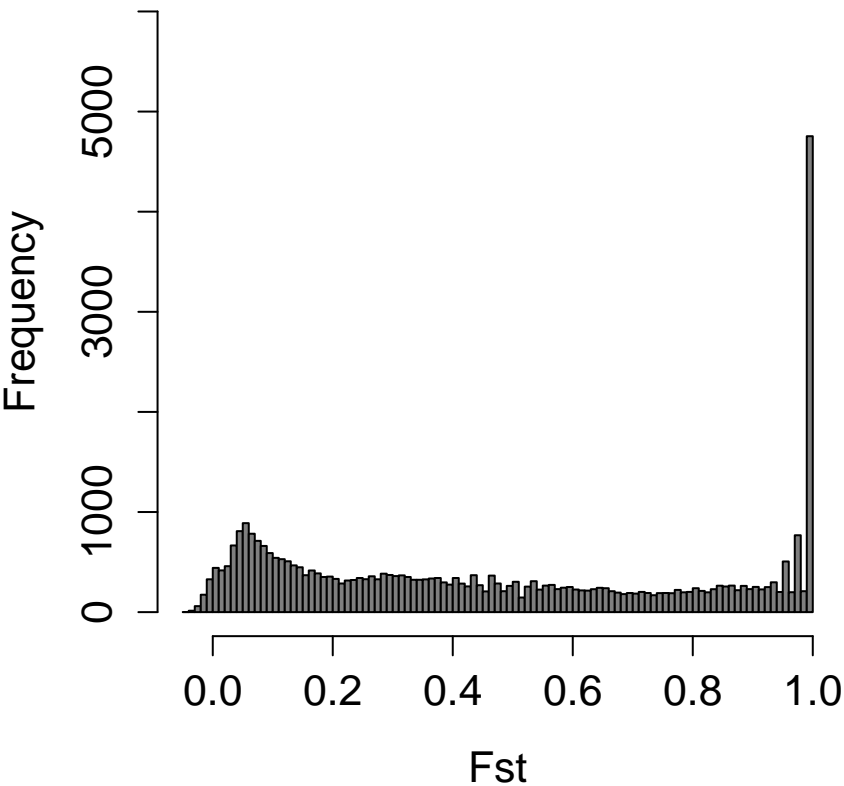

Supplement: Supplementary file 1 [file ECE3-9-14101-s001.pdf]

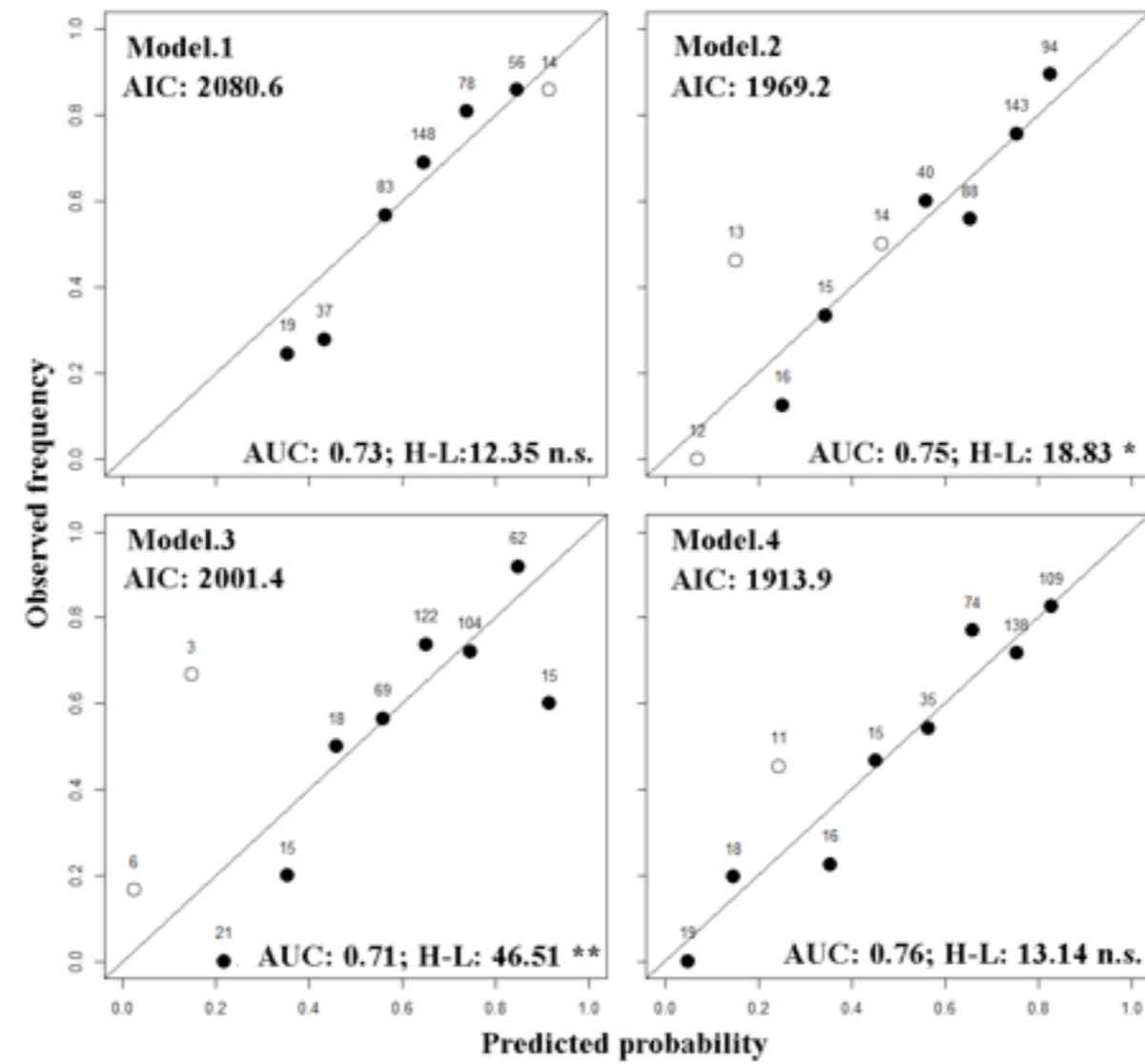

Supplement: Supplementary file 2 [file ECE3-9-14101-s002.pdf]

**Model.1**

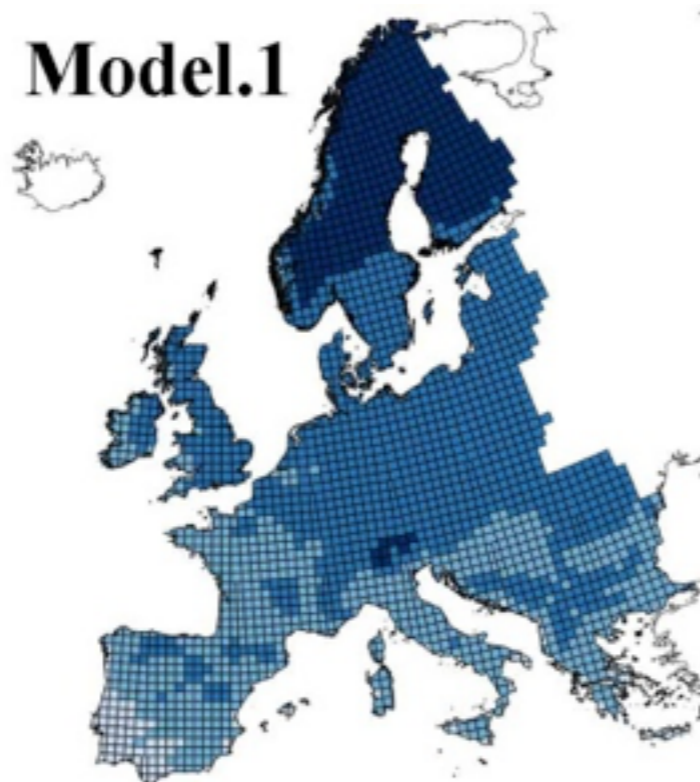

**Model.2**

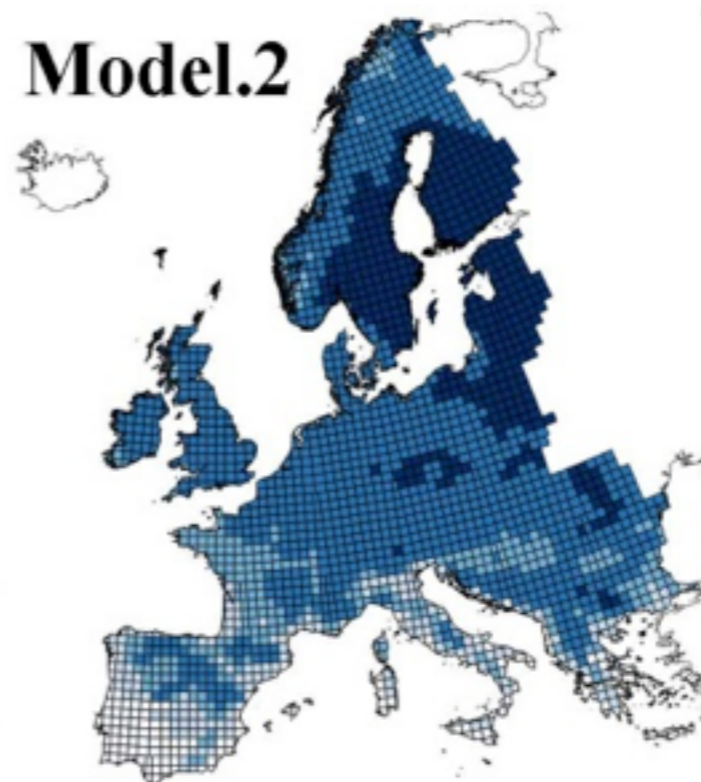

**Model.3**

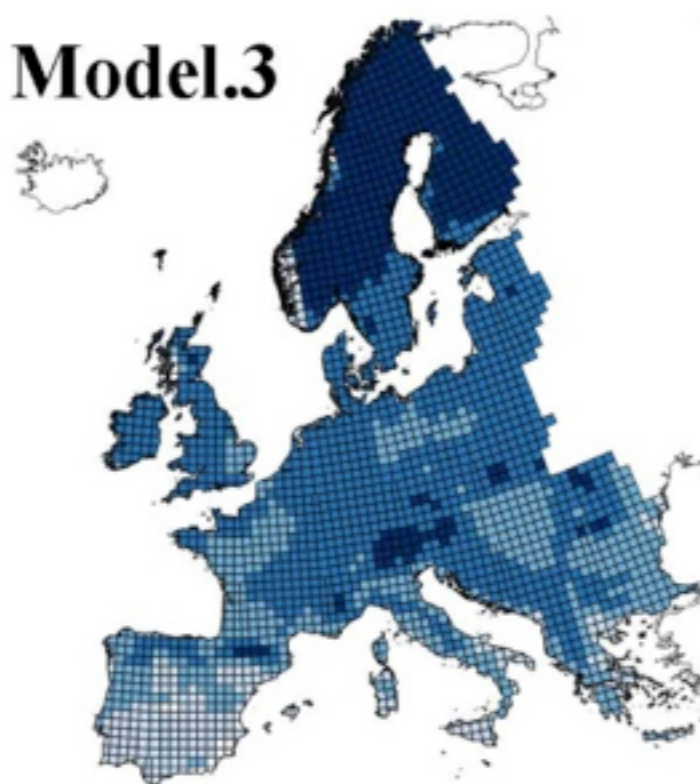

**Model.4**

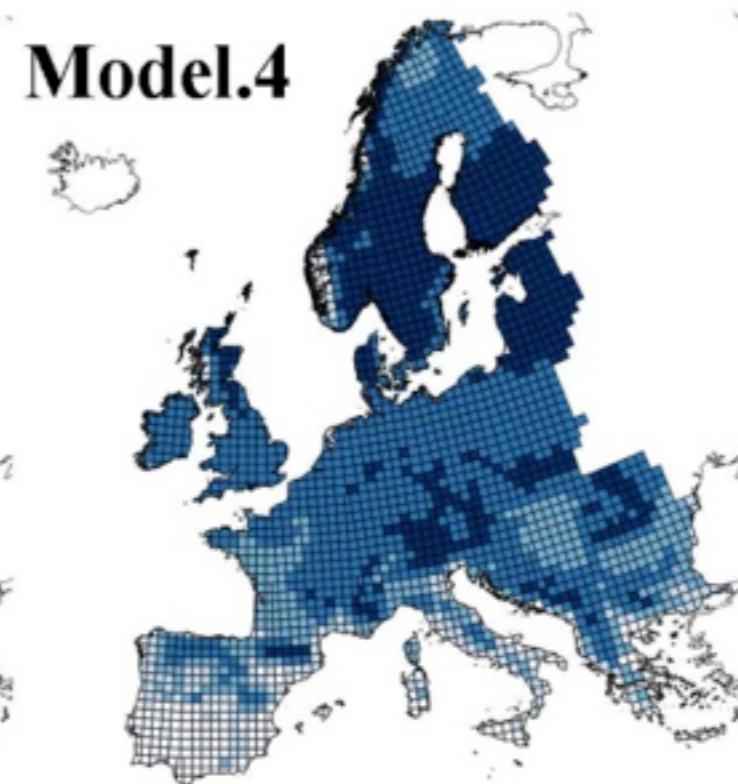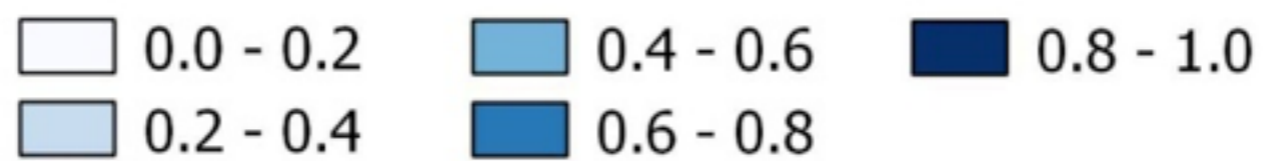

Supplement: Supplementary file 3 [file ECE3-9-14101-s003.pdf]

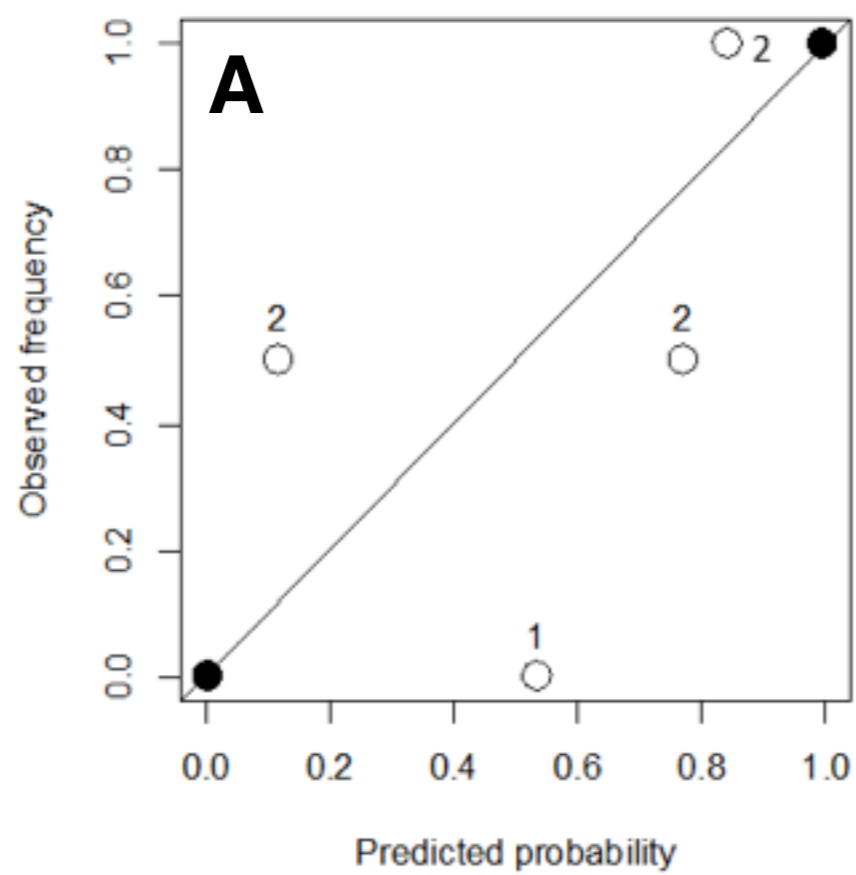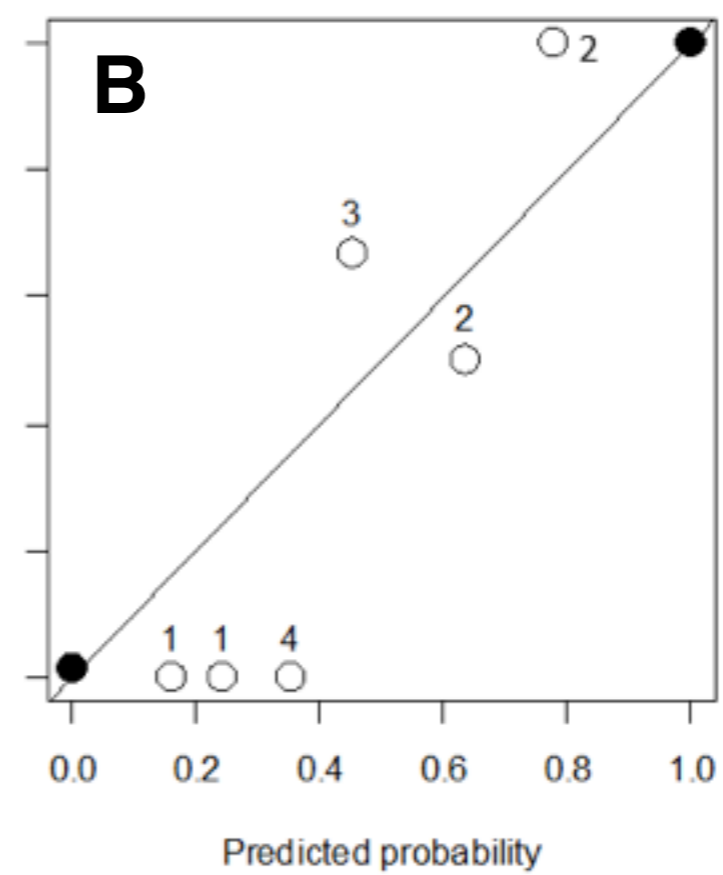

Supplement: Supplementary file 4 [file ECE3-9-14101-s004.pdf]

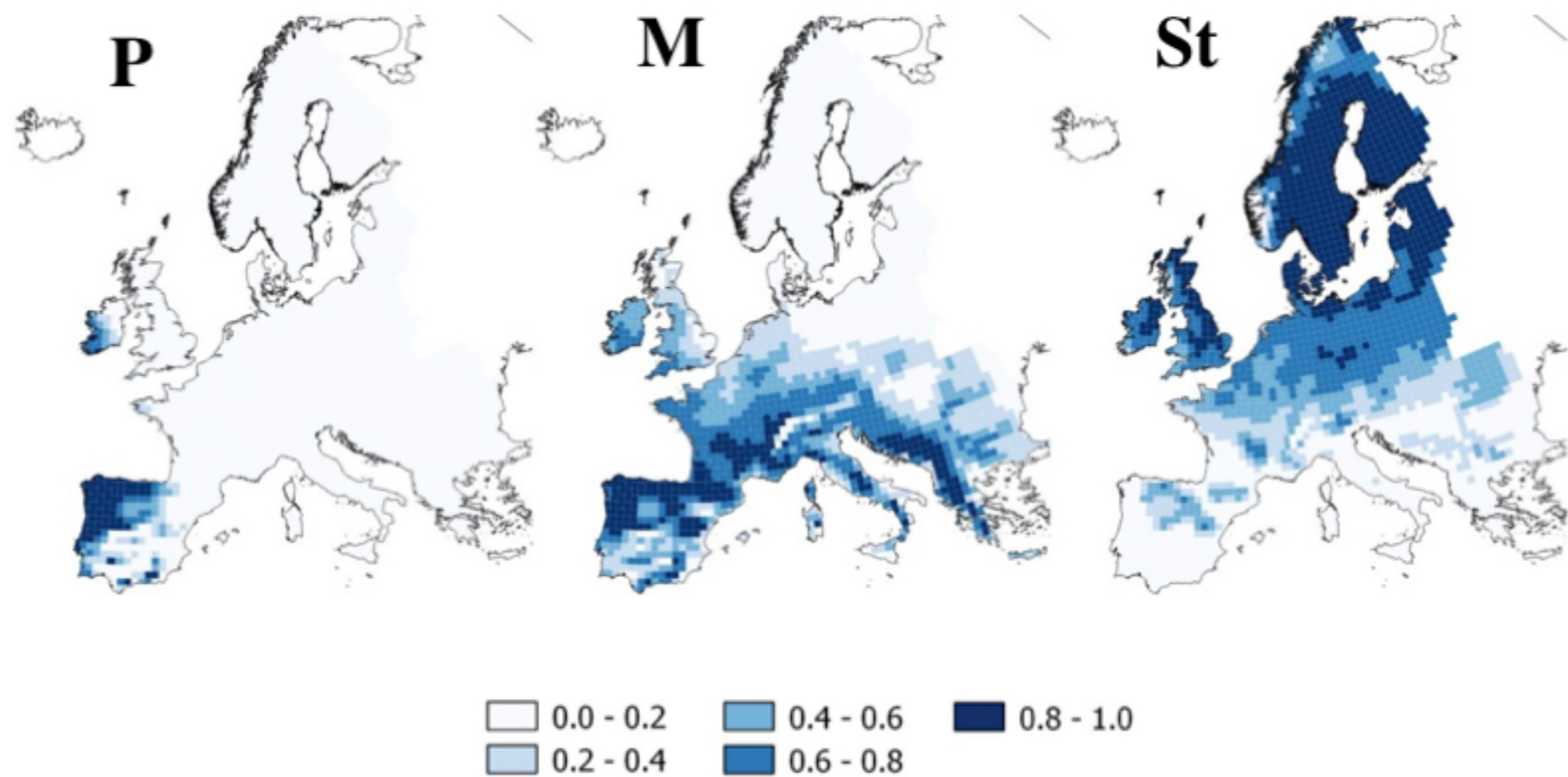

Supplement: Supplementary file 5 [file ECE3-9-14101-s005.pdf]

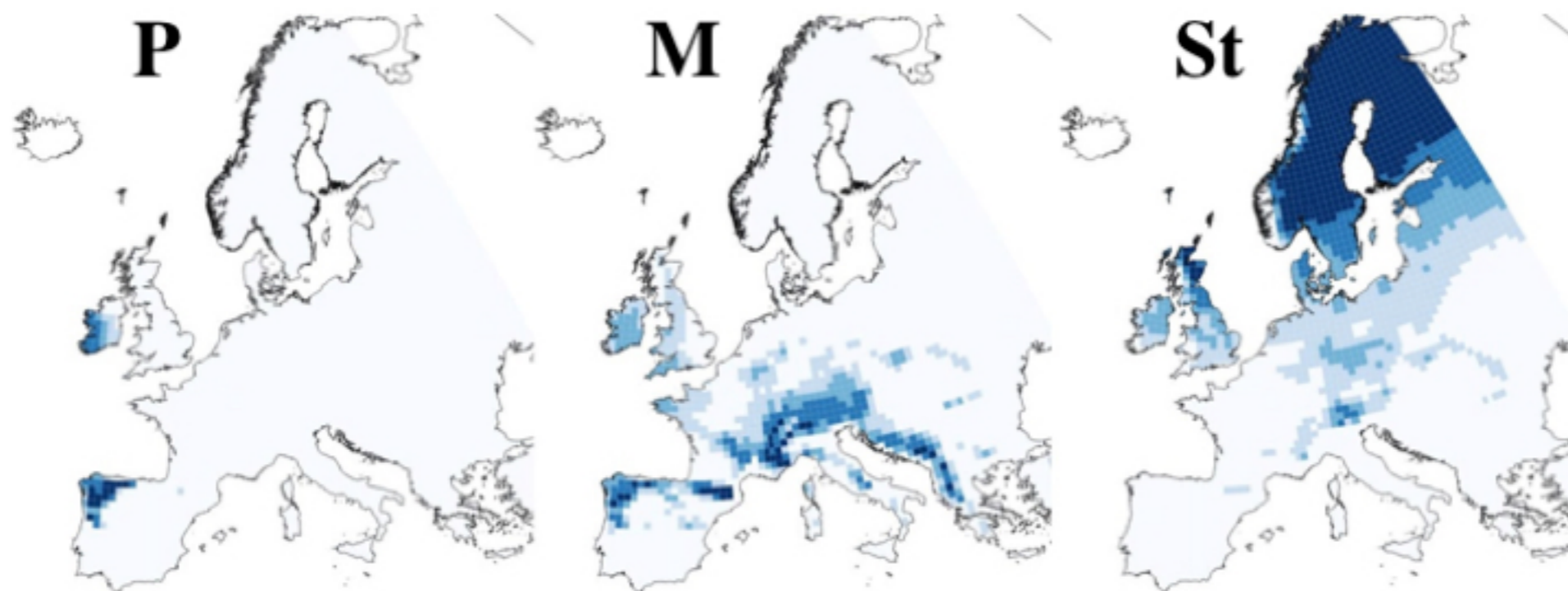

Supplement: Supplementary file 6 [file ECE3-9-14101-s006.pdf]

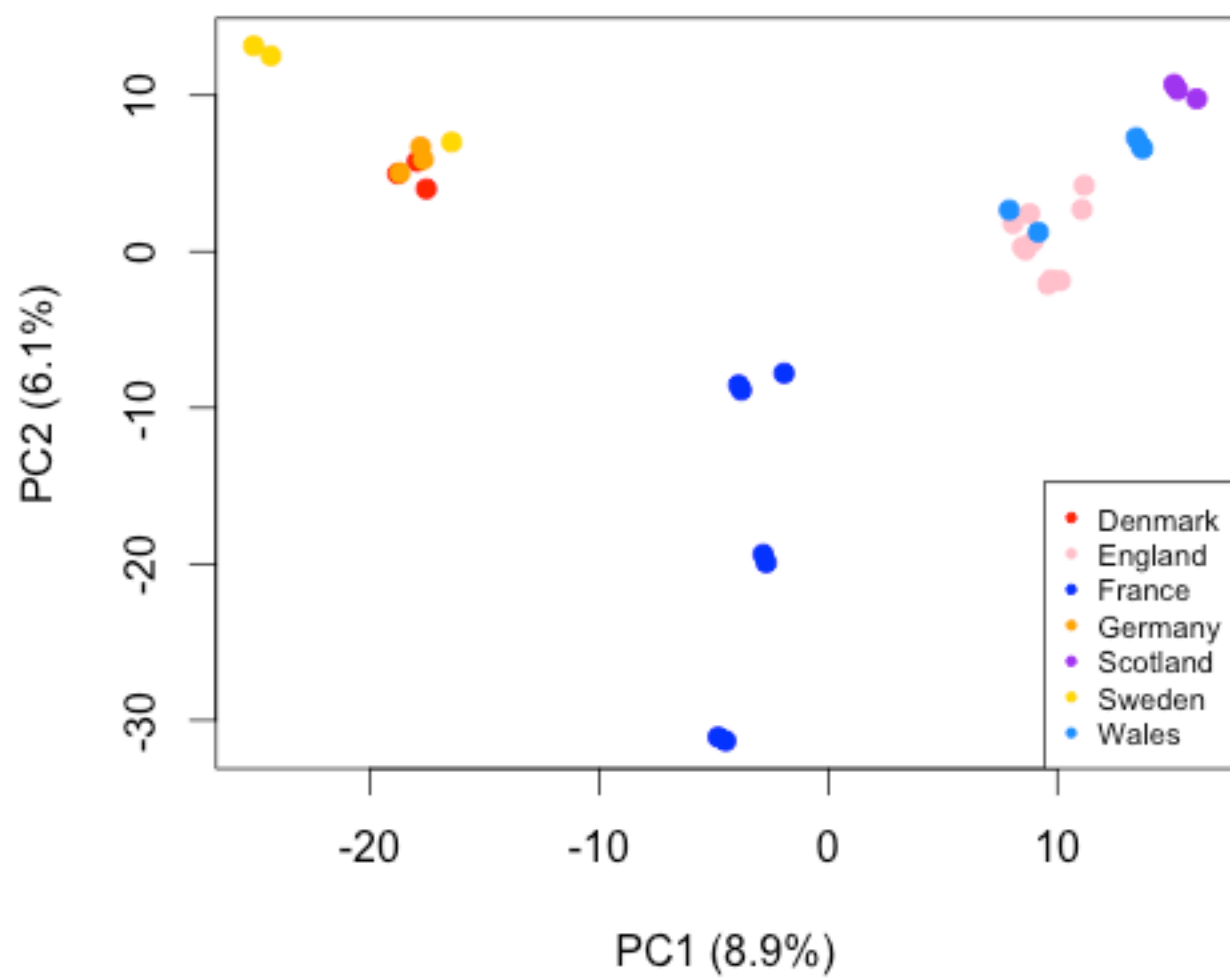

Supplement: Supplementary file 7 [file ECE3-9-14101-s007.pdf]
