# Supplementary material for: Glacial cycles drive rapid divergence of cryptic field vole species
Source: Ecol Evol. 2019 Nov 23;9(24):14101–13. doi: 10.1002/ece3.5846 (PMC6953675; doi:10.1002/ece3.5846)
Supplement: Supplementary file 11 [file ECE3-9-14101-s011.docx]

**Appendix S1**

**Figure S1:** Distribution of *F*_ST_ values for all 35,434 SNPs genotyped in the field voles (all cryptic species) studied here.

**Figure S2**: Calibration plots including information on the goodness-of-fit of the climatic niche models and their predictive performance on the validation dataset. Model.4 is the most explanatory and also has the best calibration (Hosmer and Lemeshow test (H-L), P > 0.05), lowest AIC scores, and with the highest AUC values, all indicative of a good predictive performance.

**Figure S3**: Predicted probability for occurrence of the field vole at the present time (not distinguishing between cryptic species) according to the four assessed climatic niche models.

**Figure S4**: Calibration plots showing the relationship between the predicted probability of occurrence for the climatic niche models and the observed proportion of a given cryptic species of the field vole (Portuguese, Mediterranean, and short-tailed): A) Portuguese vs Mediterranean model and B) Mediterranean vs short-tailed model. Most localities are located in the extremes of each species (*P* = 0 and 1).

**Figure S5**: Current climatic favorability for the three cryptic species of field vole in western Europe (P: Portuguese, M: Mediterranean, and St: short-tailed).

**Figure S6**: Predicted climatic distribution for Portuguese (P), Mediterranean (M), and short-tailed (St) field vole cryptic species in 2080.

Methods for Fig. S6: Model predictions suggest that ongoing climate change will negatively affect the climatic potential for the three cryptic species in western Europe. The fuzzy increment indices for the three species according to new models (Figs 4 and 6) were as follows: Portuguese: I = -0.653; Mediterranean: I = -0.566; and short-tailed: I = -0.386. That is, the climatic potential in 2080 for the Portuguese cryptic species is expected to decrease 65% of its current distribution, Mediterranean 56.6%, and short-tailed 39%. When the predicted favourabilities for 2080 are used to explore the biogeographical relationships between cryptic species, barely any areas for coexistence are predicted, only a few squares for the Portuguese and Mediterranean. The expected relationships between these species for the future differ of that obtained for present. Here, the Portuguese shows potential to displace the Mediterranean in areas of potential competition, as these are the core areas of the Portuguese cryptic species distribution. The situation for the relationship between the Mediterranean and short-tailed is quite similar to that found for present. However, for the future a potential competitive exclusion is possible in some localities of eastern Alps, where the short-tailed cryptic species could be excluded by the Mediterranean.

**Figure S7**: Intraspecific genetic structure within the short-tailed field vole using 35,434 SNP loci. Individuals are labeled by their country of origin (see legend). PCA analysis was restricted to only short-tailed field vole individuals from mainland Europe and Britain. Individuals from small offshore islands were removed to show general geographic patterns within the species.

**Table S1**: List of field vole individuals and sampling localities included in this study, ordered according to cryptic species (Portuguese, Mediterranean, and short-tailed), country of origin and location. Voucher specimens in collections of the National Museums Scotland (prefix NMS.Z), J.B. Searle tissue collection (no prefix) or CIBIO, University of Porto (prefix SM).

**Table S2**: Results of the climatic niche models explaining the current distribution range of the field vole (without distinguishing the cryptic species; coefficient / Wald test statistics; *, *P* < 0.05; **, *P* < 0.01; and ***, *P* < 0.001). BIO1: annual mean temperature and BIO12: annual precipitation as climatic predictors (Hijmans et al 2005). Squared predictors were considered in order to account for nonlinear relationships.

**Table S3**: Results of the climatic niche models discriminating between cryptic species of the field vole: Portuguese vs Mediterranean and Mediterranean vs short-tailed (coefficient / Wald test statistics; n.s., *P* > 0.05; *, *P* < 0.05; **, *P* < 0.01; and ***, *P* < 0.001). BIO3: isothermality; BIO9: mean temperature of driest quarter; and BIO15: precipitation seasonality (coefficient of variation) (Hijmans et al 2005)

Using individuals genotyped with diagnostic mtDNA and nuclear markers (*N* = 377), a model was parameterized in order to find ecogeographical gradients able to discriminate between the different cryptic species of field vole. For this purpose, two generalized models (binomial and logic link function) were performed in which the dependent variables were Portuguese vs Mediterranean and Mediterranean vs short-tailed. Bioclimatic variables (19 predictors; Hijmans et al 2005) and geographical variables (latitude and longitude) were used as predictors. A forward-backward stepwise procedure based on AIC was used to select the most parsimonious models. Table S2 shows the final models for each pair of neighbor lineages.

The predictive performance of the models was high: Portuguese vs Mediterranean (AUC: 0.999 and H-L: 5.78, *P* > 0.05) and Mediterranean vs short-tailed (AUC: 0.999 and H-L: 10.94, *P* > 0.05)(see Figure S2). These models were used to assign a given species to each 50 x 50 km UTM squares in which the field vole is present in western Europe.
